# Clinical Manifestations, Laboratory Findings, and Therapeutic Regimen in Hospitalized Children with Brucellosis in an Iranian Referral Children Medical Centre

**DOI:** 10.3329/jhpn.v31i2.16386

**Published:** 2013-06

**Authors:** Fatemeh Fanni, Leila Shahbaznejad, Babak Pourakbari, Shima Mahmoudi, Setareh Mamishi

**Affiliations:** ^1^Department of Infectious Disease, School of Medicine;; ^2^Pediatric Infectious Diseases Research Center, Tehran University of Medical Sciences, Tehran, Iran

**Keywords:** Brucellosis, Child, Diagnosis, Treatment, Iran

## Abstract

Brucellosis is considered a known widespread zoonotic disease and is endemic in Mediterranean region, like Iran. This study reviewed the clinical manifestations, laboratory findings, and therapeutic regimen in childhood brucellosis in Iran. In this retrospective study, we reviewed hospital-records of 34 consecutive children with a confirmed diagnosis of brucellosis among a total number of 10,864 patients admitted to Children's Medical Center, Tehran, Iran, between 2002 and 2010. Among the patients diagnosed with brucellosis, 22 (65%) were admitted during spring and summer. Clinical findings of these patients at admission were arthritis, splenomegaly, hepatomegaly, lymphadenopathy, maculopapular skin rashes, and fever. Anaemia (53%) and leukopenia (33%) were the most common findings in the children. Only one patient had presented with leukocytosis. Four children (12%) were thrombocytopenic, and none of patients had pancytopenia. Blood cultures were positive in 5 patients (23%). Only one patient underwent bone-marrow aspiration and had positive culture for *Brucella* spp. Positive titres were found in 33 cases (97%) in Wright test, 23 cases (96%) in Coombs test, and 16 patients (72.7%) in 2ME (2-Mercaptoethanol) test. In one case, Wright and Coombs test titres were below 1:80 while *Brucella* spp. were isolated from blood at the same time. It is concluded, prolonged fever with joint involvement and organomegaly may increase possibility of infection with *Brucella* spp. Appropriate treatment regimen by more tolerable oral drugs, with a duration of at least 8 weeks, is recommended.

## INTRODUCTION

Brucellosis or Malta fever is a known widespread zoonotic disease, and it is endemic in Mediterranean region, like Iran (1). Although Malta fever is considered an occupational disease in adults, there are several reports of childhood brucellosis in literature (1-5). This infectious disease is a common cause of fever with unknown origin (FUO) among children, particularly in endemic countries (6,7). Humans may be infected with *Brucella* spp. either due to direct contact with infected animals or ingestion of raw products of animal origin (8).

The clinical presentations of human brucellosis range from non-specific and constitutional symptoms, like prolonged fever, anorexia, or fatigue to local organ involvement, such as arthritis and neurobrucellosis (8). There is no agreement to select the best treatment regimen but long duration of treatment and prescription of more than one drug could prevent relapse of this disease (2).

The aim of the present study was to evaluate the clinical manifestations, laboratory findings, and therapeutic regimen in childhood brucellosis in an Iranian referral hospital.

### Patients and methods of selection

In this retrospective study, we reviewed hospital-records of 34 consecutive children with a confirmed diagnosis of brucellosis among a total number of 10,864 patients admitted to Children's Medical Center, Tehran, Iran, between 2002 and 2010.

The diagnosis of brucellosis was established when clinical manifestations of disease had been followed by positive serum agglutination test and isolation of *Brucella* spp. from blood or bone-marrow cultures. The cutoff titre of 1:80 in Wright and Coombs tests and 1:40 in 2ME (2-Mercaptoethanol) test were considered positive. For blood culture, 5 mL of venous blood was inoculated aseptically into the bottles consisted of trypticase soy or brain-heart infusion broth that were prepared in-house. The bottles were incubated at 37 °C for 21 days and subcultured on chocolate agar plates (9,10). *Brucella* spp. isolates were identified by Gram-staining and conventional biochemical testing. A structured checklist was prepared to record detailed demographic characteristics of patients, clinical and laboratory data, therapeutic regimen, and relapse. Organomegaly was defined by a documented assessment of abnormal size after the physical examination was performed. Relapse was defined by the presence of a clinical picture compatible with brucellosis, after a symptom-free interval following previous treatment. Data were statistically analyzed by using SPSS (version 16) software. Fisher Exact test and independent test were used in analyzing the data, and p≤0.05 was considered significant.

## RESULTS

Among 34 patients with brucellosis, 22 were male and 12 were female; mean age of the patients was 6.88 years (range 2 to 14 years). Sixteen patients (47%) were 2 to 5 years old, and 11 patients (32%) were 5 to 10 years old. Only 7 cases (20%) were between 10 and 14 years of age. Twenty-two children (65%) were admitted during spring and summer. Twenty-two cases (65%) had a history of consumption of raw or unpasteurized milk products; 5 (15%) had direct contact with domestic animals; and in 7 (20%), the route of transmission remained unclear. There was no significant difference between children's gender and route of infections. Nine patients (26%) had previous family history of brucellosis, and 7 cases (20%) had been hospitalized for FUO.

Major complaints and symptoms of patients were fever (n=25, 74%), anorexia (n=24, 71%), fatigue (n=23, 68%), and arthralgia (n=22, 65%). Other symptoms included sweating in 20 (59%), weight loss in 17 (50%), abdominal pain in 8 (24%), vomiting and headache each in 7 (21%), sore throat in 5 (15%), cough in 4 (14%), and epistaxis in 1 (3%).

Clinical findings at admission were arthritis (n=26, 77%), splenomegaly (n=11, 32%), hepatomegaly (n=8, 24%), lymphadenopathy (n=6, 18%), and skin rashes (n=1, 3%). The joints most-commonly affected were the hip and knee, followed by elbow, wrist, ankle, and sacroiliac joints.

According to reference ranges for laboratory tests and procedures (11), haematologic and serologic findings are listed in the table. Anaemia (53%) and leukopenia (33%) were the most common findings in the children in our study; only one patient had presented with leukocytosis. Four children (12%) were thrombocytopenic, and none of the patients had pancytopenia.

The erythrocyte sedimentation rate (ESR) was considered high (more than 30 mm in one hour) in 13 cases (38%). Examination for C-reactive protein (CRP) was performed in 30 patients by quantitative method, and it was positive in 19 cases (63%).

Among 16 patients who had liver function test (ALT: Alanine aminotransferase, AST: Aspartate aminotransferase) found in their documents, only 3 (19%) had ALT level above normal range, and high AST level was reported in 5 (31%) patients.

Wright test was performed for all cases (34 patients) but Coombs and 2ME tests were done for 24 and 22 cases respectively. According to cutoff points, positive titres were found in 33 cases (97%) in Wright test, 23 cases (96%) in Coombs, and 16 patients (72.7%) in 2ME test. The highest Wright, Coombs Wright, and 2ME titres were found in one patient, including 1:5120, 1:10240, and 1:640 respectively. In one case, Wright and Coombs Wright titres were below 1:80 (1:40) but *Brucella* spp. were isolated from blood at the same time that could be due to acute phase of disease. Blood cultures were performed in 22 cases and were positive in 5 patients (23%). Only one patient underwent bone-marrow aspiration and had positive culture for *Brucella* spp. The species of *Brucella* in patients with positive bacterial culture was *Brucella melitensis*.

In 21 children (62%) younger than 8 years, a combination of trimethoprim-sulphamethoxazole and rifampicin was prescribed. Other 13 patients (38%) who were older than 9 years were treated with doxycycline and rifampicin. Duration of treatment was 8 weeks for both groups, and all children tolerated these combinations for 8 weeks. Most patients became symptoms-free at the end of the 7^th^ day after initiation of the treatment. The patients were hospitalized until clinical improvement was achieved. During follow-up, the check-ups were done once a month, and then every 3-6 months. In our study, only one patient among 34 cases experienced relapse because of early interruption of treatment, and others did not have any symptoms or signs of infection during the routine outpatient follow-up period.

**Table.** Laboratory findings in brucellosis cases

Laboratory examination	Normal reference values 9	No. and % of patients
Leukocyte count: ×1,000 cells/mm^3^	1-4 year(s): 6-17.5	Leukopenia: 11 (33%)
	4-8 years: 5.5-15.5	Normal: 22 (64%)
	8-13 years: 4.5-13.5	Leukocytosis: 1 (3%)
Haemoglobin: g/dL	1-6 year(s): 10.5-14	
	6-12 years: 11.5-15.5	Anaemia: 18 (53%)
	12-18 years (Girl): 12-16	Normal: 16 (47%)
	12-18 years (Boy): 13-16	
Platelet count: 1,000 cells/mm^3^	150-400	Thrombocytopenia: 4 (12%)
		Normal: 27 (80%)
		Thrombocytosis: 3 (9%)
ESR	Less than 30	Normal: 21 (62%)
		Elevated: 13 (38%)
CRP	Less than 5 mg/L	Negative: 11 (37%)
No. of patients: 30		Positive: 19 (63%)
ALT (unit/L)	1-19 year(s): 5-45	Normal: 13 (81%)
No. of patients: 16		Elevated: 3 (19%)
AST (unit/L)	1-9 year(s): 15-55	Normal: 11 (69%)
No. of patients: 16	9-19 years: 5-45	Elevated: 5 (31%)

## DISCUSSION

In this study, 34 children were diagnosed as brucellosis patients in an eight-year period. In comparison with previous reports from this centre (1988-2001), the incidence of brucellosis remained unchanged over the years (nearly 3-4 patients per year) (12). Although our hospital is a referral centre and our patients were from all over the country, the incidence of brucellosis is still higher in agricultural regions, like North of Iran. Roushan and his colleagues reported 111 childhood brucellosis in a 4-year period (1999-2003) in Babol, a city in the north of Iran, (approximately 27 cases per year) (1).

Sex distribution for brucellosis in our study is similar to others, in which majority of childhood brucellosis cases were boys (1,2,4,12,13). There are some ideas to explain this male predominance; boys more than girls may have contact with animals and consume insecure foods but we found no significant difference between boys and girls in the route of infection.

Almost half of the patients in our study were 2 to 5 years old. It was in contrast to the findings of Tanir *et al*. (2) from Turkey and Giannakopoulos and colleagues (4) from Greece that nearly 18% of children with brucellosis were younger than 5 years. In their studies, half of the patients were from farmer families and rural areas, and occupational contact with animals was unavoidable; so, older children might be at increasing risk of contact with the infected animals. In our study, majority of children were infected by dairy products; so, younger kids tend to become ill more.

The majority of patients were admitted during spring and summer. Consumption of dairy products, like ice cream and travelling to villages in these seasons, increase the risk of brucellosis in children (1,12) but, in adults, incidence of this disease is more frequent during winter and spring in lambing seasons (8,14).

In endemic regions, like Iran, positive family history of brucellosis, consumption of unpasteurized dairy products or contact with domestic animals may lead clinicians to investigate and diagnose brucellosis in febrile children.

Clinical manifestation of brucellosis is often ambiguous, making the diagnosis difficult. Fever and arthralgia were the most prevalent complaint in children suffering from brucellosis (3,4,8,13-15) as reported in adults (3,14,16). Bone and joint involvement, including peripheral arthritis, sacroilitis, and spondylitis, may occur in brucellosis but peripheral arthritis is non-erosive and particularly common in children and young adults (17,18). In our study, similar to other reports, hips and knees were affected most frequently (15,19).

Less frequent symptoms, such as fatigue, sweating, weight loss, abdominal pain, and headache, were reported in previous studies (1-4,12,13,15). Organomegaly, including hepatomegaly and/or splenomegaly, were found in nearly one-third of children with brucellosis in our study. The frequency of organomegaly is very different in literature (1-4,12,13,15). *Brucella* spp. involve reticuloendothelial system, and organomegaly is frequently found during examination but this broad range of reports may be due to examiners’ experience, quality of physical examination, and duration of the disease (4).

Anaemia, thrombocytopenia, and leukopenia are common during infection with *Brucella* spp. (1,2,5,12,13,15,20-24). Although there was no patient with pancytopenia in our study, there are some reports of pancytopenia in other studies (5,20-24).

Different elevated levels of ESR and CRP are predictable during any infections, like brucellosis. In some studies, 38% to 87% of patients with brucellosis had elevated ESR, and 34% to 81% of them had positive values of CRP (1,2,12,13). Because of technical problems in culturing *Brucella* spp., the rate of positive blood culture in brucellosis is very different and varies from 18% to 42% (2,4,12,13).

There are several treatment regimens for brucellosis. In adults, tetracycline is recommended for therapy but this class of antibiotics is typically avoided in children aged ≤8 years due to the risk of dental staining (25). The preferred regimen in children below 8 years of age is rifampicin plus trimethoprim-sulphamethoxazole for 8 weeks plus gentamicin 5 mg/kg/day for the first 5 days (26). There is strong evidence that the tetracycline produces rapid relief of symptoms but ≥6 weeks of treatment is required (27,28).

In the multicentre retrospective study of childhood brucellosis in Chicago, no relapses or treatment failure occurred in children whose initial therapy included rifampicin or those who were administered three-drug regimens (25).

The optimal duration of therapy remains unclear. Sufficient duration of therapy and avoiding monotherapy are considered important rules in reducing relapse rate, the condition that is not rare in brucellosis. It has been reported that inappropriate antibiotic therapy may be associated with higher relapse rates (29). In some studies, lower level of relapse was reported with combination of at least 2 drugs in 8-week therapy compared to 6 weeks (1,12). Roushan *et al*. reported treatment of children with trimethoprim-sulphamethoxazole, and rifampicin for 8 weeks had higher cure rate when compared with 6 weeks (30).

### Conclusions

Prolonged fever with joint involvement and organomegaly may increase possibility of infection with *Brucella* spp.; appropriate treatment regimen by more tolerable oral drugs with duration of at least 8 weeks, is recommended.
